# Molecular Determinants of Magnolol Targeting Both RXRα and PPARγ

**DOI:** 10.1371/journal.pone.0028253

**Published:** 2011-11-29

**Authors:** Haitao Zhang, Xing Xu, Lili Chen, Jing Chen, Lihong Hu, Hualiang Jiang, Xu Shen

**Affiliations:** State Key Laboratory of Drug Research, Shanghai Institute of Materia Medica, Chinese Academy of Sciences, Shanghai, China; Semmelweis University, Hungary

## Abstract

Nuclear receptors retinoic X receptor α (RXRα) and peroxisome proliferator activated receptor γ (PPARγ) function potently in metabolic diseases, and are both important targets for anti-diabetic drugs. Coactivation of RXRα and PPARγ is believed to synergize their effects on glucose and lipid metabolism. Here we identify the natural product magnolol as a dual agonist targeting both RXRα and PPARγ. Magnolol was previously reported to enhance adipocyte differentiation and glucose uptake, ameliorate blood glucose level and prevent development of diabetic nephropathy. Although magnolol can bind and activate both of these two nuclear receptors, the transactivation assays indicate that magnolol exhibits biased agonism on the transcription of PPAR-response element (PPRE) mediated by RXRα:PPARγ heterodimer, instead of RXR-response element (RXRE) mediated by RXRα:RXRα homodimer. To further elucidate the molecular basis for magnolol agonism, we determine both the co-crystal structures of RXRα and PPARγ ligand-binding domains (LBDs) with magnolol. Structural analyses reveal that magnolol adopts its two 5-allyl-2-hydroxyphenyl moieties occupying the acidic and hydrophobic cavities of RXRα L-shaped ligand-binding pocket, respectively. While, two magnolol molecules cooperatively accommodate into PPARγ Y-shaped ligand-binding pocket. Based on these two complex structures, the key interactions for magnolol activating RXRα and PPARγ are determined. As the first report on the dual agonist targeting RXRα and PPARγ with receptor-ligand complex structures, our results are thus expected to help inspect the potential pharmacological mechanism for magnolol functions, and supply useful hits for nuclear receptor multi-target ligand design.

## Introduction

Nuclear receptors are ligand-regulated transcription factors, involving multiple signalling pathways, among which RXRα and PPARγ are in the central positions. RXRα plays its role in diverse physiological processes, including cell development, apoptosis, and homeostasis [1], [2]. And it predominantly expresses in liver, kidney, epidermis and intestine [3]. RXRα agonists have been found to exhibit glucose-lowing, insulin-sensitizing, as well as anti-obesity effects [4]. For example, LGD1069, which is approved for the treatment of cutaneous T-cell lymphoma, also shows decreased fasting plasma glucose and insulin in *ob/ob* mice [5]. While another RXRα agonist LG100268 exhibits its efficiency in reducing fasting plasma glucose and improving insulin resistance [6]. Thus, RXRα agonists have great potentials for the treatment of metabolic diseases.

PPARγ distributes in adipose tissue, regulating adipocyte differentiation, lipid storage, inflammation, hypertension, and atherosclerosis [7]. It has favourable effects on glucose uptake, lipid metabolism and energy expenditure. Moreover, its activation promotes adipogenesis and insulin sensitivity [8]. PPARγ agonists are reported to exhibit a variety of pharmacological potentials in anti-hyperglycemia, anti-hyperinsulinemia, and lowering triglycerides in adipose, muscle and liver [9]. Thiazolidinediones (TZDs) targeting PPARγ, such as Rosiglitazone and Pioglitazone, have been approved to improve insulin sensitivity. Considering the undesirable side effects of TZDs [9], a new type of chemical compounds with therapeutic properties but different from TZDs are in urgent needs.

Once activated by their agonists, RXRα and PPARγ translocate into the nucleus forming RXRα:RXRα homodimer or RXRα:PPARγ heterodimer, which subsequently binds to RXRE or PPRE to initial their target genes transcription, respectively [10]. Recently, there are increasing numbers of reports on the synergistic effects of RXRα and PPARγ agonists. As indicated, coactivation of RXRα and PPARγ exhibits enhanced efficiencies in the metabolism of glucose and lipid [11], as well as the inhibition of cancer cell migration and invasiveness [12]. Combined treatment with RXRα and PPARγ agonists also inhibit nitric oxide and tumor necrosis factor-alpha production in rat Kupffer cells [13], and suppress proliferation of immortalized endometrial stromal cells [14]. All these facts have thus addressed the pharmacological significances of RXRα and PPARγ coactivation by their agonists. However, the dual agonist that binds and activates both RXRα and PPARγ has not been reported by far.

**Figure 1:**
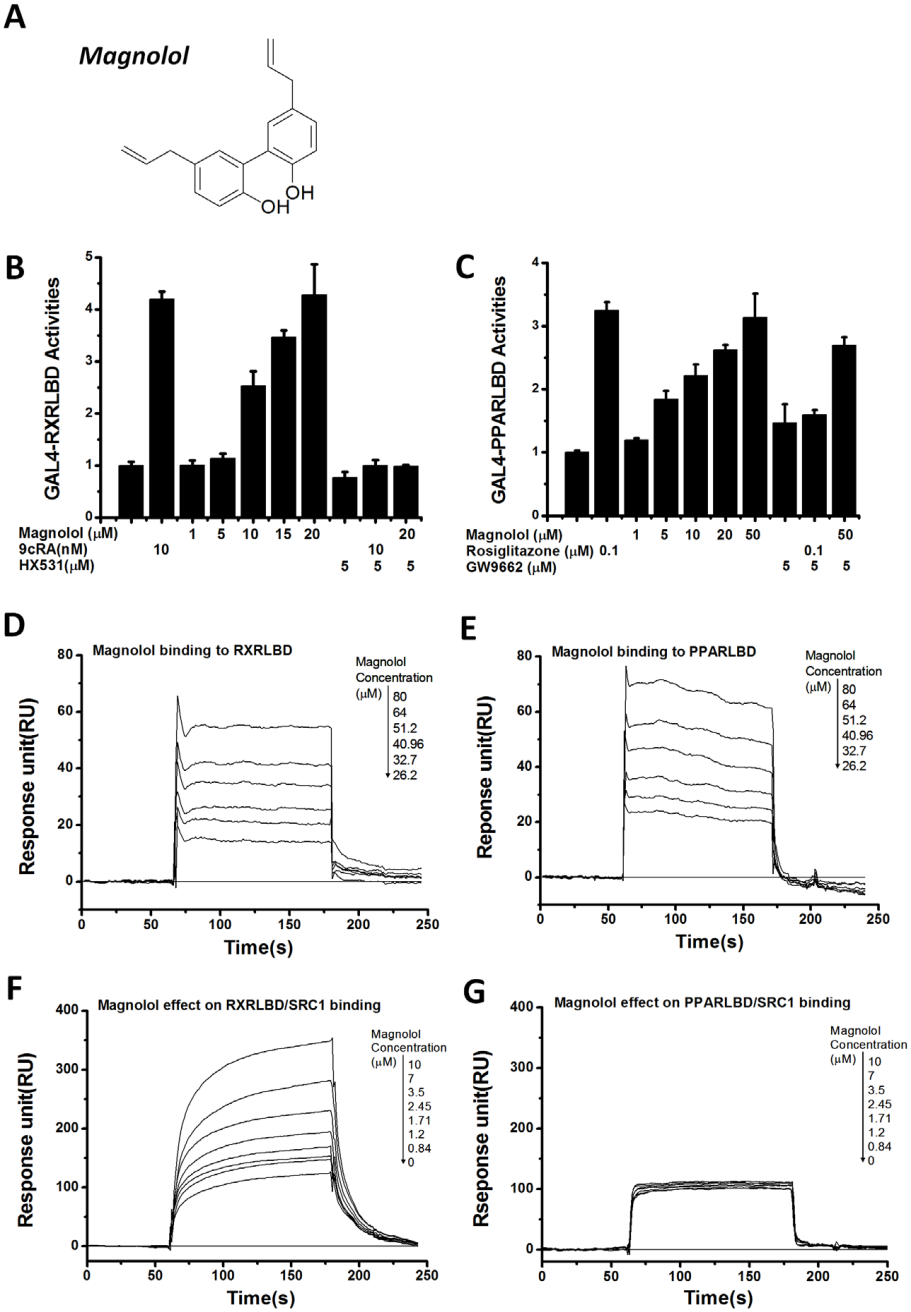
Magnolol as a dual agonist of RXRα and PPARγ. (**A**) Chemical structure of magnolol. (**B–C**) Magnolol dose-dependently activated the transcription of GAL4DBD-RXRαLBD (**B**) and GAL4DBD-PPARγLBD (**C**) in HEK-293T cells, which could be suppressed by RXRα antagonist HX531 and PPARγ antagonist GW9662, respectively. RXRα agonist 9-*cis*-retinoic acid (9*c*RA) and PPARγ agonist Rosiglitazone were used as positive controls. (**D–E**) Magnolol dose-dependently bound to RXRαLBD (**D**) and PPARγLBD (**E**) in SPR technology based assays. (**F–G**) Magnolol dose-dependently enhanced SRC1 recruitment to RXRαLBD (**F**), instead of PPARγLBD (**G**) in SPR technology based assays.

In the current work, we screen our house in-lab library of natural products for RXRα and PPARγ agonists. Interestingly, we find that magnolol is a dual agonist of both RXRα and PPARγ. Magnolol (5,5′-diallyl-2,2′-dihydroxybiphenyl, **Figure 1A**) is one of the main constituents from the stem bark of *Magnolia officinalis*, which is used in the traditional Chinese medicine to cure cough, diarrhea and allergic rhinitis [15]. Magnolia bark was also suggested to be effective in combating metabolic syndrome [16]. Treatment with magnolol decreased fasting blood glucose and plasma insulin levels, and prevented the pathological complications in type 2 diabetic rats [17]. Remarkably, magnolol was reported to enhance adipocyte differentiation and glucose uptake in 3T3-L1 adipocyte cells [18] and prevent the development of diabetic nephropathy [17]. Moreover, the high glucose-induced TGFβ1 and fibronectin expressions were inhibited by magnolol *via* ERK/MAPK/Akt signalling pathway in human retinal pigment epithelial cells under diabetic conditions [19], while the anti-oxidative and hepatoprotective effects of magnolol were shown on liver damage in rats [20].

Although magnolol can bind and activate both RXRα and PPARγ, the transactivation results shows biased agonism of magnolol to induce the transcription of PPRE mediated by RXRα:PPARγ heterodimer, instead of RXRE mediated by RXRα:RXRα homodimer. To reveal the molecular basis for magnolol function, we determine the crystal structures of both RXRαLBD-magnolol and PPARγLBD-magnolol. Based on these two complex structures, we find that magnolol adopts surprising binding modes on RXRα and PPARγ with key interactions for magnolol agonism determined. Therefore, our results are expected to not only shed light on the potential pharmacological application for magnolol, but also supply useful hits for multi-target drug design based on the nuclear receptors.

## Results and Discussion

In the discovery of new ligands from the lab in-house natural products library against RXRα and PPARγ, we construct a screening platform based on in-cell mammalian one hybrid assays. Among the natural products with the activities to activate either RXRα or PPARγ, magnolol unexpectedly shows its agonistic functions on both of these two nuclear receptors, with EC_50_ values of 10.4 µM and 17.7 µM, respectively (**Figure 1B and C**). Additionally, the magnolol-induced RXRα and PPARγ activations can be suppressed by the known RXRα and PPARγ antagonists HX531 and GW9662, respectively (**Figure 1B and C**), implying that magnolol takes its effects by targeting both of these two nuclear receptors. We further perform surface plasmon resonance (SPR) technology based experiments to detect the physical binding of magnolol to the purified RXRαLBD and PPARγLBD. As indicated in **Figure 1D and E**, magnolol dose-dependently binds to RXRαLBD and PPARγLBD with *K*_D_ values of 45.7 µM and 1.67 µM, respectively.

As nuclear receptors, RXRα and PPARγ need to recruit their coactivators to initiate the transcription of target genes [4]. Thus we further investigate whether magnolol can enhance these two nuclear receptors binding to the common coactivator steroid receptor coactivator-1 (SRC1) using SPR based technology. As indicated in **Figure 1F**, magnolol can increase RXRαLBD-SRC1 interactions in a dose-dependent manner. However, this natural product exhibits no effect on SRC1 recruitment to PPARγLBD (**Figure 1G**). Considering there are many other coactivators for PPARγ function [7], magnolol may probably take its effect by recruiting other coactivator instead of SRC1 for PPARγ involved transcription.

**Figure 2:**
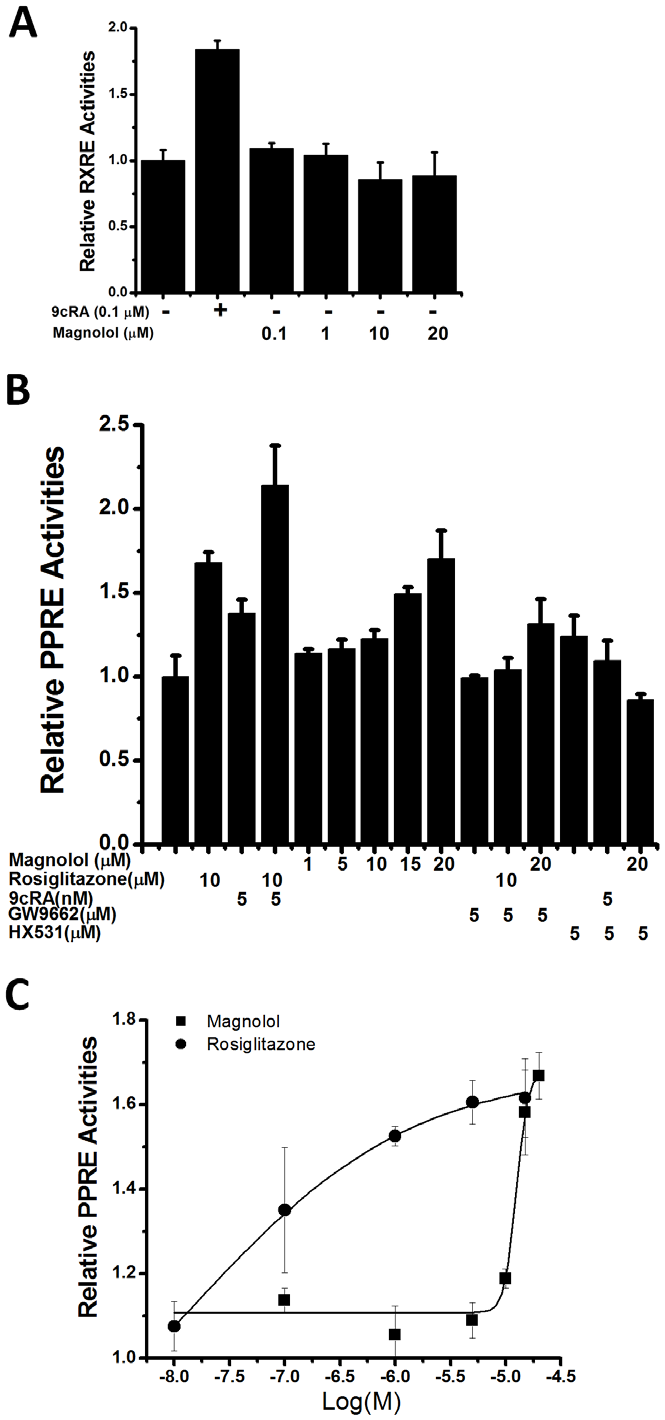
Magnolol as a biased agonist on PPRE transcription. (**A–B**) Magnolol could not activate the transcription of RXRE mediated by RXRα:RXRα homodimer (**A**), while activating the transcription of PPRE mediated by RXRα:PPARγ heterodimer in a dose-dependent manner (**B**). RXRα agonist 9*c*RA, RXRα antagonist HX531, PPARγ agonist Rosiglitazone, and PPARγ antagonist GW9662 were used as controls. (**C**) Activating curves of magnolol and Rosiglitazone on PPRE transcription indicated that magnolol was a PPARγ full agonist, although magnolol exhibited lower activities in their lower concentrations.

In activation of the downstream genes transcription, RXRα and PPARγ have to form RXRα:RXRα homodimer and RXRα:PPARγ heterodimer binding to their response elements. Thus we further evaluate the effects of magnolol on the activities of RXRα:RXRα homodimer and RXRα:PPARγ heterodimer using transactivation analyses on their response elements RXRE and PPRE. As indicated in **Figure 2A and B**, magnolol induces the transcription of PPRE in a dose-dependent manner. However, this compound exhibits no activity on RXRE transcription. Moreover, the magnolol-effect on PPRE transcription can be suppressed by both RXRα and PPARγ antagonists HX531 and GW9662, respectively (**Figure 2B**), which is in good accordance to our in-cell mammalian one hybrid assays (**Figure 1B and C**). It thus indicates that magnolol binding to both RXRα and PPARγ is required to activate PPRE transcription. Additionally, magnolol exhibits lower activities in their lower concentrations, compared to PPARγ agonist Rosiglitazone (**Figure 2C**). However, magnolol surprisingly shows equal activities to Rosiglitazone in their high concentrations, indicating magnolol is a PPARγ full agonist (**Figure 2C**). In conclusion, we identify magnolol from the natural product library functioning as a dual agonist of both RXRα and PPARγ, with the biased transcriptional activity on PPRE instead of RXRE.

**Figure 3:**
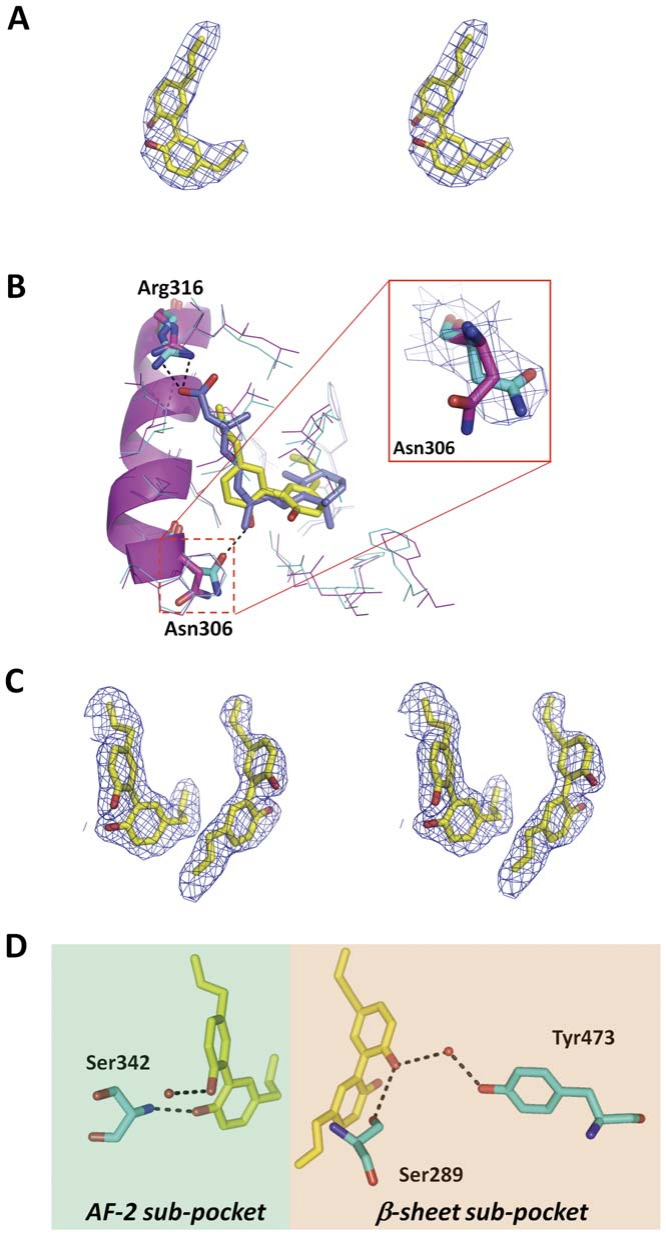
Crystal structures of RXRαLBD-magnolol-SRC1 and PPARγLBD-magnolol. (**A**) Electron density of magnolol bound into RXRα ligand-binding pocket in stereo view (contoured at 1.0σ level). (**B**) Comparison of receptor-ligand interactions between 9*c*RA-bound and magnolol-bound RXRαLBDs. 9*c*RA (in blue sticks) formed hydrogen bonds with Arg316 (in magenta sticks) in the C-terminus of helix 5 (in magenta ribbon), while magnolol (in yellow sticks) formed hydrogen bonds with Asn306 (in cyan sticks) in the N-terminus of helix 5. Density map around Asn306 was shown in the right to indicate its conformational changes. All other hydrophobic residues involving 9*c*RA (in magenta lines) or magnolol interactions were the same (shown in cyan lines). (**C**) Electron density map of magnolol bound into PPARγ ligand-binding pocket in stereo view (contoured at 1.0σ level). (**D**) The two magnolol molecules formed hydrogen bonds with Ser342 in β-sheet, Tyr473 in AF-2 motif, and Ser289 in helix 3 of PPARγ, as well as water-mediated hydrogen bonds.

As indicated in the previously reported crystal structures of RXRα ligand-binding domain complex with agonists, the essential activation function-2 (AF-2) motif in RXRα exhibits significant conformational changes. AF-2 motif overturns itself to cover the ligand-binding pocket upon agonist binding, thus exposing the surface for recruiting the coactivator SRC1 and initializing the transcription of target genes [21], [22], [23]. The typical chemical structure of RXRα agonist consists of the acidic and hydrophobic moieties to adapt the L-shaped ligand-binding pocket of RXRα [5], [6]. Different from previously reported RXRα agonists, magnolol possesses two identical 5-allyl-2-hydroxyphenyl moieties. Thus we wonder how magnolol functions as an agonist of RXRα. To reveal the molecular basis for magnolol binding and activating RXRα, we determine the crystal structure of RXRαLBD-magnolol complex with SRC1 coactivator peptide. Magnolol-bound RXRαLBD exhibits a dimeric packing of RXRα. The electron density around magnolol is shown in **Figure 3A**. Magnolol binds into the hydrophobic ligand-binding pocket, and induces conserved conformational changes of AF-2 motif for SRC1 coactivator peptide recruitment. Magnolol is found to adapt itself to an L-shaped conformation, with two 5-allyl-2-hydroxyphenyl moieties occupying each side of the L-shaped pocket, respectively. The typical RXRα agonists always form a hydrogen bond with Arg316 in the C-terminus of helix 5 [5], [6]. However, magnolol uses one hydroxyl group to form a hydrogen bond with Asn306 in the N-terminus of helix 5 (**Figure 3B**). Such an interaction induces an overturning of Asn306, compared with the known agonist 9-*cis*-retinoic acid-bound RXRαLBD structure (**Figure 3B**). Moreover, helix 3 is observed to bend towards the ligand-binding pocket from its position in apo RXRαLBD structure, which is consistent with the known agonist-bound RXRαLBD structures [5], [6]. Therefore, from our determined crystal structure of RXRαLBD-magnolol-SRC1, the agonist magnolol employs a distinct binding mode for RXRα activation by interacting with Asn306 in the N-terminus of helix 5, instead of Arg316 in the C-terminus of helix 5. And magnolol adapts its two 5-allyl-2-hydroxyphenyl moieties occupying the hydrophobic and acidic sides of the pocket, respectively.

Different from RXRα with the L-shaped ligand-binding pocket, PPARγ uses a much larger Y-shaped pocket for ligand-binding [24]. And PPARγ ligand-binding pocket can be divided into two sub-pockets, AF-2 sub-pocket and β-sheet sub-pocket [24]. PPARγ agonists are categorized as full and partial agonists, depending on their activities in the cell-based reporter assays [25]. It is suggested that PPARγ partial agonists bind only β-sheet sub-pocket, while full agonists always occupy both AF-2 and β-sheet sub-pockets to activate PPARγ [26]. Magnolol is determined to be a full agonist of PPARγ in the current work (**Figure 2C**). Thus we wonder how magnolol binds such a Y-shaped pocket for PPARγ activation. In our determined crystal structure of PPARγLBD-magnolol, the electron density map around magnolol is shown in **Figure 3C**. Interestingly, two magnolol molecules are found in PPARγ ligand-binding pocket, one in AF-2 sub-pocket and the other in β-sheet sub-pocket. The hydroxyl group of magnolol in AF-2 sub-pocket forms a hydrogen bond with Ser289 in helix 3, as well as water-mediated hydrogen bonds with Tyr473 in AF-2 motif (**Figure 3D**). Direct interactions between agonist and AF-2 motif are believed to play a crucial role in the conformational changes of PPARγ AF-2 motif, and surface formation for coactivator recruitment [26]. On the other side, the hydroxyl group of magnolol in β-sheet sub-pocket interacts with Ser342 in β-sheet with a hydrogen bond (**Figure 3D**). Moreover, there is also a water-mediated hydrogen bond with magnolol in β-sheet sub-pocket to further stabilize the ligand binding (**Figure 3D**). Our findings have thus revealed an unexpected binding mode of magnolol on PPARγ, with two identical chemical compounds binding two different sub-pockets, which probably lead for new PPARγ agonists design.

To evaluate the degree of cooperativity of the two magnolol molecules binding to PPARγ, Hill coefficient is determined. The value of approximately 2 indicates that magnolol binding is positively cooperative, and both the binding sites can bind magnolol simultaneously. Thus two magnolol molecules cooperatively induce PPARγ activation by interacting with both AF-2 motif and β-sheet, respectively. Furthermore, the fact that two magnolol molecules cooperatively bind to PPARγ also explains the reason why magnolol exhibits lower activities on PPRE transcription, compared to PPARγ agonist Rosiglitazone (**Figure 2C**). Although magnolol and Rosiglitazone are both PPARγ full agonists, their transactivation curves indicate their different mechanisms (**Figure 2C**). Only one molecule of Rosiglitazone is necessary for PPARγ activation, while two magnolol molecules are required to bind PPARγ. Considering that the magnolol-effect on PPRE transcription can also be suppressed by RXRα antagonist HX531, and HX531 can inhibit RXRα agonist 9*c*RA activity on PPRE, it thus suggests that magnolol binding to RXRα is also necessary for PPRE transcription. Therefore, totally three magnolol molecules are required for PPRE transcription, with one molecule binding to RXRα and two molecules binding to PPARγ.

Magnolol was once characterized as a PPARγ agonist with the computer aided modelling [27]. However, our co-crystal structure of PPARγLBD-magnolol reveals a distinct ligand binding mode. As indicated in **Figure 3D**, magnolol in AF-2 sub-pocket is found to form not only a hydrogen bond with Ser289 in helix 3, but also water-mediated hydrogen bonds with Tyr473 in AF-2 motif. On the other side, in β-sheet sub-pocket of PPARγ, magnolol interacts with Ser342 in β-sheet (**Figure 3D**), instead of Gly284 that was determined by the computer aided modelling. Moreover, we also find a water-mediated hydrogen bond with magnolol in β-sheet sub-pocket to further stabilize the ligand binding (**Figure 3D**). Considering that the water-mediated interactions within PPARγLBD-magnolol is still delicate to be determined by the computer based modelling, our co-crystal structure is expected to supply further insights into the future computer based modelling.

Honokiol, an analogue of magnolol, shares some certain biological properties with magnolol [28]. And honokiol was reported to have anti-angiogenic, anti-inflammatory and antitumor functions, but the mechanisms of honokiol actions are still elusive. Here we find that magnolol targets both RXRαLBD and PPARγLBD, thus how honokiol interacts with these two nuclear receptors will be of potentially important and interesting. Moreover, knowledge of mechanisms of magnolol and honokiol actions may assist novel synthetic analogues development in the future.

**Figure 4:**
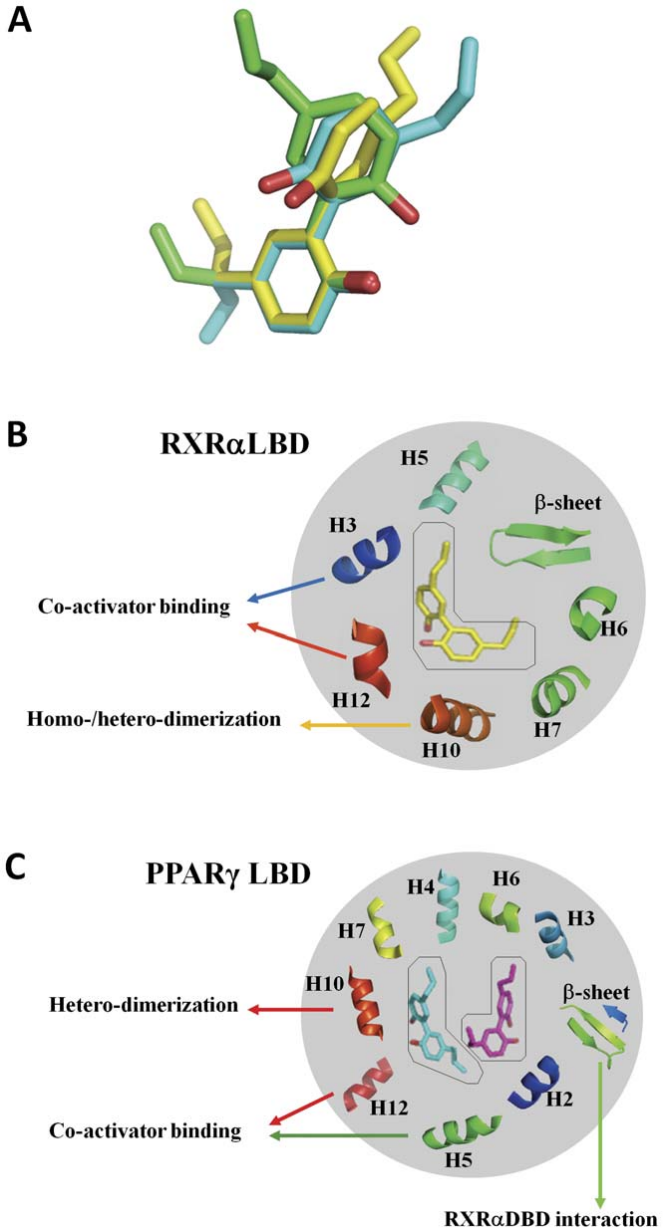
Key interactions for magnolol function on RXRα and PPARγ. (**A**) Magnolol exhibited three different conformations upon binding into RXRα and PPARγ ligand-binding pockets. Magnolol in RXRα ligand-binding pocket was shown in yellow, while the two magnolol molecules in PPARγ ligand-binding pocket were shown in green and cyan, respectively. (**B–C**) Secondary structures with which magnolol interacted were shown in both RXRα (**B**) and PPARγ (**C**) ligand-binding pockets. The functions of these secondary structures in the coactivator recruitment, homo-/heterodimerization and DNA-binding domain (DBD) interactions were indicated.

From the RXRαLBD-magnolol and PPARγLBD-magnolol structures, it is suggested that the hydroxyl groups of magnolol play essential roles in the receptor-ligand interactions. In RXRαLBD-magnolol structure, the hydroxyl group of magnolol contacts with Asn306 in helix 5 of RXRα. While, in PPARγLBD-magnolol structure, the hydroxyl groups from the two bound ligands interact with Ser342 in β-sheet, Tyr473 in AF-2 motif, and Ser289 in helix 3 of PPARγ, respectively. Additionally, magnolol adopts surprising binding modes on these two nuclear receptors. Although magnolol is big enough to accommodate mostly the L-shaped RXRα ligand-binding pocket, two magnolol molecules have to cooperatively occupy the much larger Y-shaped PPARγ ligand-binding pocket. Furthermore, the single bond connecting the two 5-allyl-2-hydroxyphenyl moieties of magnolol endows this chemical compound flexibility to fit the different pocket sizes of RXRα and PPARγ. As shown in **Figure 4A**, magnolol molecules exhibit three different conformations when it binds to RXRα and PPARγ. **Figure 4B and C** show the key secondary structures of RXRα and PPARγ, with which magnolol makes direct interactions. Our findings are in good accordance with that the homo-/heterodimeric interface and coactivator binding surface of RXRα and PPARγ are critical for both of these two nuclear receptors activation. And all of these secondary structures of RXRα and PPARγ are conserved in the agonist binding and interactions. Considering the large differences between RXRα L-shaped pocket and PPARγ Y-shaped pocket, future dual agonist design may focus on PPARγ sub-pockets, since each PPARγ sub-pocket has a similar size to the whole pocket of RXRα. The agonist which can accommodate to RXRα ligand-binding pocket and the two PPARγ sub-pockets with preferred activities will probably have potentials to activate both of these two nuclear receptors.

## Materials and Methods

### Luciferase assays

Mammalian one hybrid and transactivation experiments were performed using luciferase assays in HEK293T (human embryonic kidney) cells (obtained from ATCC). Transient transfection was conducted using Lipofectamine 2000 (Invitrogen) according to the manufacturer's guideline. For the mammalian one hybrid tests for RXRα or PPARγ, UAS-TK-Luc reporter plasmid was co-transfected with GAL4DBD-RXRαLBD or GAL4DBD-PPARγLBD. For the transactivation assays of RXRE or PPRE, pGL3-RXRE-Luc was co-transfected with pcDNA3.1-RXRα, or pGL3-PPRE-Luc was co-transfected with both pcDNA3.1-RXRα and pcDNA3.1-PPARγ. Cells were incubated with varied concentrations of compounds for 24 h. The known RXRα agonist 9-*cis*-retinoic acid (9*c*RA), RXRα antagonist HX531, PPARγ agonist Rosiglitazone, and PPARγ antagonist GW9662 were used as controls. All compounds were purchased from Sigma, dissolved in DMSO, and prepared to different concentrations. Luciferase activities were then measured using Dual Luciferase Assay System kit (Promega).

### Protein expression and purification

The coding sequence of human RXRαLBD (residues 221–458) was cloned to the vector pET15b, and E. coli strain BL21 (DE3) was used for protein expression. The culture was induced with 0.5 mM IPTG and incubated at 25°C for 6 hours. His-tagged RXRαLBD was purified with Ni-NTA resin (Qiagen) and the tag was then removed by Thrombin (Novagen). The protein was further purified with Superdex 200 (Amersham Pharmacia Biotech).

The coding sequence of human PPARγLBD (residues 204–477) was cloned to the vector pGEX6P-1. GST-PPARγLBD was expressed with 0.2 mM IPTG at 18°C for 6 hours. GST-tag was removed by PreScission protease (GE Healthcare). The protein was further purified with Superdex 200 (Amersham Pharmacia Biotech).

The SRC-1 coactivator peptide was commercially synthesized with the sequence KHKILHRLLQDSS.

### Surface plasmon resonance (SPR) technology based assays

Binding affinities of magnolol towards purified RXRαLBD and PPARγLBD were analyzed using Biacore 3000 instrument (GE Healthcare). Proteins were covalently immobilized to CM5 chip using a standard amine-coupling procedure in 10 mM sodium acetate buffer (pH 4.2). The chip was equilibrated with a continuous flow of running buffer (10 mM HEPES, pH 7.4, 150 mM NaCl, 3 mM EDTA, 0.005% (v/v) surfactant P20) for 2 hours. Subsequently, magnolol in a gradient of concentrations were injected into the channels at a flow rate of 20 µL/min for 60 seconds, followed by disassociation for 120 seconds. For the coactivator SRC1 recruitment assays, biotin-labelled SRC1 was immobilized to SA chip. Different concentrations of magnolol were incubated with 5 µM RXRαLBD or PPARγLBD for 1 hour, and then injected to the channel at a flow rate of 20 µL/min for 60 s, followed by disassociation for 120 s.

### Crystallization

All crystallization experiments were performed by hanging-drop method at 20°C. RXRαLBD was mixed with SRC-1 coactivator peptide and magnolol in a ratio of 1∶3∶5. Crystals grew in the condition of 100 mM Tris, pH 7.5, 20% PEG3350. For the PPARγLBD-magnolol complex, the ratio of PPARγLBD:magnolol was 1∶5. Crystals grew in the condition of 4 M sodium formate.

### Data collection and structure determination

Diffraction data was collected at BL17U of Shanghai Synchrotron Radiation Facility in China, and integrated with HKL2000 [29]. Phasing and refinement were carried out with Refmac5 [30]. Model building was manually performed with COOT [31]. The statistics of the data collection and structure refinement were summarized in Table 1. Atomic coordinates and structure factors of RXRαLBD-magnolol-SRC1 and PPARγLBD-magnolol have been deposited to Protein Data Bank under accession codes 3R5M and 3R5N.

**Table 1:** Data collection and refinement statistics.

	RXRαLBD-magnolol-SRC1	PPARγLBD-magnolol
**Data collection**		
Space group	*P*2_1_ 2_1_ 2_1_	*P*4_3_ 2_1_ 2
Cell dimensions		
*a, b, c* (Å)	65.95, 65.83, 110.29	66.04, 66.04, 155.26
*α, β, γ* (°)	90, 90, 90	90, 90, 90
Resolution (Å)	32.9−2.8 (2.85−2.80)[a]	40.0−2.0 (2.07−2.00)
R_sym_ or R_merge_	0.056 (0.381)	0.053 (0.398)
*I*/*σI*	11.8 (2.7)	51.8 (7.2)
Completeness (%)	98.8 (99.7)	94.2 (90.7)
Redundancy	3.6 (3.6)	5.9 (6.3)
**Refinement**		
Resolution (Å)	32.9−2.8	40.0−2.0
No. reflections	11 863	20 400
R_work_/R_free_	0.249/0.292	0.188/0.213
No. atoms	3 678	2 194
*B*-factors	47.1	41.0
R.m.s. deviations		
Bond lengths (Å)	0.008	0.006
Bond angles (°)	1.103	0.968
Ramachandran plot (%)		
Most favored regions	95.5	98.0
Allowed regions	4.5	2.0

[a] Values in parenthesis are for highest resolution shell.
